# Effect of Sintering Temperature on Microstructure Characteristics of Porous NiTi Alloy Fabricated via Elemental Powder Sintering

**DOI:** 10.3390/ma17030743

**Published:** 2024-02-04

**Authors:** Tianhu Miao, Sha Zhan, Xiaojuan Chen, Li Hu

**Affiliations:** 1 School of Materials Science and Engineering, Chongqing University of Technology, Chongqing 400054, China; mth@stu.cqut.edu.cn (T.M.); zhans@stu.cqut.edu.cn (S.Z.); cxj@2020.cqut.edu.cn (X.C.); 2 Chongqing Key Laboratory of Die Technology, Chongqing University of Technology, Chongqing 400054, China

**Keywords:** porous NiTi alloy, powder metallurgy, sintering temperature, microstructure characteristics

## Abstract

To investigate the effect of the sintering temperature on the microstructure characteristics of porous NiTi alloys, two types of porous NiTi alloys with equal atomic ratios were fabricated via elemental powder sintering at 950 °C and 1000 °C. Afterwards, optical microscopy (OM), scanning electron microscopy (SEM) and transmission electron microscopy (TEM) were collectively applied to investigate the pore characteristics and microstructure of the fabricated porous NiTi alloy. The results show that when the sintering temperature increases from 950 °C to 1000 °C, the average pore size increases from 36.00 μm to 181.65 μm, owing to the integration of these newly formed small pores into these pre-existing large-sized pores. The measured density increases from 2.556 g/cm^3^ to 3.030 g/cm^3^, while the porosity decreases from 60.4% to 51.8%. This is due to the occurrence of shrinkage after the sufficient diffusion of atoms. Furthermore, the characterization results confirm that a change in the sintering temperature would not change the phase types within a porous NiTi alloy; namely, the matrix consists primarily of B2 NiTi, with a significant amount of Ni_4_Ti_3_ precipitates and a small amount of Ni_3_Ti precipitates and Ti_2_Ni precipitates. However, as the sintering temperature increases, the number of Ni_4_Ti_3_ precipitates decreases significantly. The formation of a Ni_4_Ti_3_ phase in the present study is closely related to the enrichment of Ni content in the matrix owing to the diffusion rate difference between Ni atoms and Ti atoms and the absence of a transient liquid phase (TLP) during the sintering process owing to the relatively low sintering temperature (lower than the eutectic temperature). Moreover, the increasing sintering temperature speeds up the atom diffusion, which contributes to a reduction in the enrichment of Ni as well as the number of formed Ni_4_Ti_3_ precipitates.

## 1. Introduction

NiTi alloys have great development potential in many fields, such as the automobile industry, aerospace and intelligent manufacturing and, in particular, in the field of biomedicine due to their ideal biocompatibility, excellent damping performance, unique shape memory effect and superelasticity [1,2]. However, when used for human implantation, a dense NiTi alloy still faces challenges such as poor bonding with bone tissue and a mismatch in elastic modulus, resulting in stress shielding and, ultimately, implantation failure [3,4,5]. Research has demonstrated that the incorporation of a pore structure in dense NiTi alloys can effectively tackle the aforementioned challenges. By possessing a pore structure, porous NiTi alloys not only retain the exceptional mechanical properties of their dense counterparts but also facilitate the growth of bone tissue in external implants. This leads to a stronger bond at the interface between the implant and the bone compared to dense NiTi alloys [6,7]. Therefore, porous NiTi alloys exhibit promising development prospects in the field of biomedicine.

Numerous researchers have recently developed porous NiTi alloys using various methods and conducted corresponding studies. Chen et al. [8] investigated the fabrication of porous NiTi alloys through a space-controlled self-propagating (SCSP) method. After appropriate powder treatment, they observed a consistent pore distribution in the fabricated alloys, which consist entirely of B2 NiTi, along with excellent superelasticity. Yang et al. [9] examined the compression deformation behaviors of porous NiTi alloys with small pillar thicknesses (ranging from 0.2 mm to 0.6 mm) produced using the selective laser melting (SLM) method. The study also explored the influence of scanning speed, ranging from 200 mm/s to 800 mm/s, on the mechanical properties. A maximum compressive strain of 28% was achieved in a porous NiTi alloy with a strut thickness of 0.4 mm using a scanning speed of 400 mm/s. In a study by Lai et al. [10], porous NiTi alloys were fabricated through the microwave sintering (MWS) method using a magnesium (Mg) space holder. These experimental results revealed that at sintering temperatures equal to or below 900 °C, the fabricated porous NiTi alloys predominantly consisted of B2 NiTi, with only a minor presence of B19’ NiTi. As the sintering temperature increases, the porosity of the fabricated porous NiTi alloys decreases, and the compressive strength initially increases, reaching its maximum value at 900 °C before declining. Ismail et al. [11] proposed a novel technique for manufacturing porous NiTi alloys through a metal injection molding (MIM) method with the assistance of transient liquid phase (TLP) sintering. The fabricated porous NiTi alloy possesses a high porosity of 40%, which is aided in the early stages by a TLP and then by Kirkendall diffusion into a percolating network of particles. Zhu et al. [12] fabricated a porous NiTi alloy via elemental powder sintering (PS). They reported that the pressing pressure has an obvious influence on the density and porosity of the manufactured porous NiTi alloy, which would further affect the ultimate compressive strength and the flexural strength of the porous NiTi alloy. Meanwhile, they found that when the sintering temperature is above 942 °C, the strength of the porous NiTi alloy increases remarkably.

However, the abovementioned investigations mainly focus on illuminating the effects of processing parameters on the porosity and comprehensive mechanical properties of fabricated porous NiTi alloys. Investigations about the effect of sintering temperature (which is termed a key processing parameter) on the pore characteristics and microstructure characteristics of fabricated porous NiTi alloys have not been specifically and systematically reported. To obtain an in-depth understanding of the correlation between the sintering temperature and their pore characteristics and microstructure, porous NiTi alloy samples with a Ni/Ti composition of 50:50 (at.%) were prepared using the elemental PS method in an argon atmosphere, with NaCl serving as the space holder, in the present study. Subsequently, the pore characteristics and microstructure of fabricated porous NiTi alloys were examined using optical microscopy (OM), scanning electron microscopy (SEM) and transmission electron microscopy (TEM).

## 2. Materials and Methods

The adopted raw materials consisted of Ti powder (purity: ≥99.95%; average particle size: ≤50 μm, Beijing Gaoke New Materials Technology Co., Ltd., Beijing, China), carbonyl Ni powder (purity: ≥99.9%; average particle size: ≤8 μm, Shanghai Naiou Nano Technology Co., Ltd., Shanghai, China) and NaCl powder (purity: AR; average particle size: ≤500 μm, Wuxi Jingke Chemical Co., Ltd., Wuxi, China). Specifically, the adopted Ti powder was produced by the hydrogenation–dehydrogenation method, and the carbonyl Ni powder and Ti powder were weighed in an atomic ratio of 50:50. The volume fraction of NaCl powder was equal to the sum of the volumes of Ti powder and carbonyl Ni powder. The reason for choosing NaCl powder as the space holder material in the present study is that Bansiddhi et al. [13] have proven that NaCl powder can provide sufficient stability to withstand pressure and temperature while fabricating porous NiTi alloys. The specific preparation process has been well documented in [14]; therefore, these contents will not be depicted here. To illuminate the influence of the sintering temperature on the pore characteristics and microstructure characteristics of the fabricated porous NiTi alloys, the chosen sintering temperatures in the present study included 950 °C and 1000 °C, and the holding time was set to 4 h. 

The X-ray diffraction (XRD) system PANalytical Empyrean Series 2 (Cu-Kα radiation λ = 0.154 nm, Malvern Panalytical Ltd., Malvern, UK) was applied to identify the phase composition of the initial powders. The operating voltage, scanning range and scanning rate were set at 40 KeV, 20–90° and 2°/min, respectively. An optical metallographic microscope (DMI5000M) was used to investigate the pore morphology of the fabricated porous NiTi alloys, which were sintered at 950 °C and 1000 °C. Samples for OM characterization were first ground using various SiC papers (#400, #800 and #1200) and then etched in a solution containing 50 vol.% H_2_O, 40 vol.% HNO_3_ and 10 vol.% HF. Nano Measure 1.2 software was then applied in the present study to analyze the pore size of the fabricated porous NiTi alloys. To guarantee the statistical validity of the results, three different locations were tested. Furthermore, the porosity and density were determined using the Archimedes drainage method on an FA5003J electronic density analysis balance at eight different locations within the fabricated porous NiTi alloys. Furthermore, the characteristics of powders and the microstructure of the fabricated porous NiTi alloys were examined using a JCM-7000 SEM system (JEOL Ltd., Tokyo, Japan) and a FEI NOVA 400 Zeiss Sigma field emission SEM system (FEI Corporation, Hillsboro, OR, USA). Both of them were equipped with an energy dispersive spectrometer (EDS). Samples for SEM characterization were initially prepared by mechanical grinding, followed by electrochemical polishing in an electrolyte containing 20 vol.% of sulfuric acid and 80 vol.% of ethanol. The applied SEM polishing voltage, current and temperature were ~35 V, ~40 mA and ~15 °C, respectively. The identification of the second phase within the fabricated porous NiTi alloys was performed using TEM on an FEI Tecnai G^2^ instrument (operating voltage: 300 KeV, FEI Corporation, Hillsboro, OR, USA). TEM samples were firstly prepared by mechanical grinding to a thickness of ~70 μm, followed by electrochemical thinning to about 20 nm in an electrolyte containing 60 vol.% of CH_3_OH, 34 vol.% of C_4_H_10_O and 6 vol.% of HClO_4_. The applied TEM polishing voltage, current and temperature were ~20 V, ~35 mA and ~−25 °C, respectively.

## 3. Results and Discussion

### 3.1. Characterization of Initial Powders and Mixed Powder

**Figure 1:**
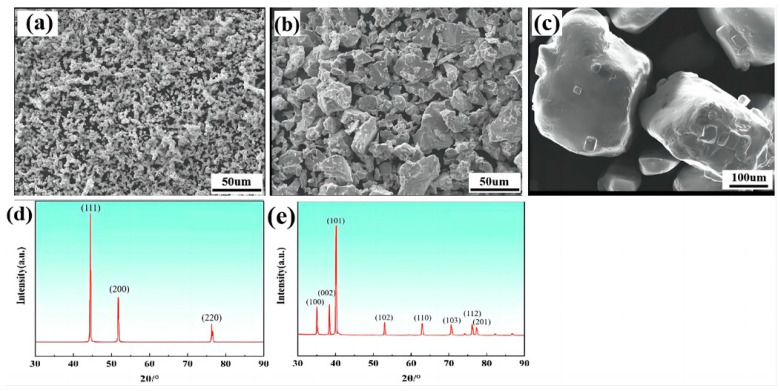
Characterization results of initial powders: (**a**) carbonyl Ni powder; (**b**) Ti powder; (**c**) NaCl powder; (**d**) XRD peaks of carbonyl Ni powder; and (**e**) XRD peaks of Ti powder.

Figure 1a illustrates the morphology of the original carbonyl Ni powders, which exhibit an approximately spherical shape with an average particle size ranging from 5 to 8 μm. Although the particle size distribution is relatively uniform, these particles tend to agglomerate with each other. The XRD analysis in Figure 1d reveals the presence of three diffraction peaks at 2 θ angles of about 44.49°, 51.84° and 76.37°. They correspond to the crystal planes (111), (200) and (220) of Ni with a face-centered cubic crystal structure, respectively. In Figure 1b, the original Ti powders are depicted. These particles display irregular shapes, with a highly uneven particle size distribution of ≤50 µm. The XRD analysis in Figure 1e shows three diffraction peaks at 2θ angles of about 35.09°, 38.39° and 40.16°, respectively. These peaks correspond to the crystal planes (100), (002) and (101) of Ti with a hexagonal close-packed crystal structure, respectively. Figure 1c shows the particle morphology of the original NaCl powders. These particles appear roughly prismatic in shape, with an irregular particle size distribution ranging from 200 to 500 μm.

**Figure 2:**
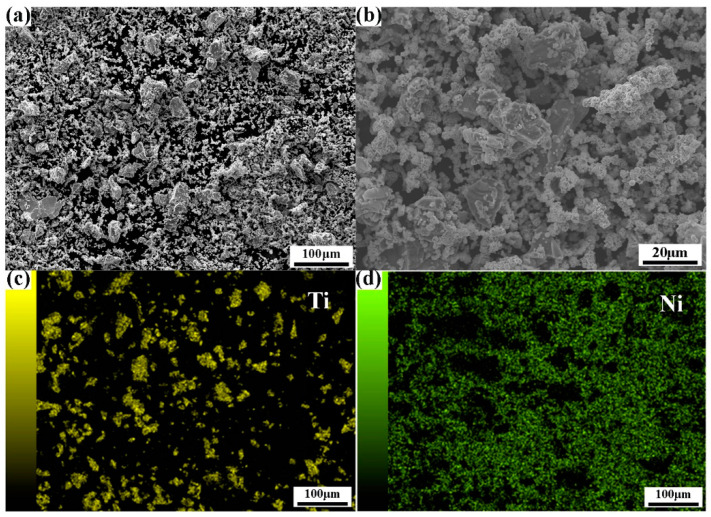
Characterization of mixed powder after ball milling for 4 h: (**a**,**b**) SEM images at different magnifications; (**c**,**d**) element distribution maps via EDS.

Uniform powder mixing is a crucial step in the preparation process, which would significantly impact the sintering quality of the fabricated samples. Deng et al. [15] reported that if the mixing time is not sufficient, larger agglomerated particles may persist, leading to denser interparticle bonding. After pressing and sintering, the porosity and pore size of these agglomerated regions may be lower compared to non-agglomerated regions. In the present study, the duration of ball milling was chosen to be 4 h, and the experimental results for the mixed powder are presented in Figure 2. The distribution of mixed powder at different SEM magnifications shows an overall uniform distribution in Figure 2a,b. The element distribution in Figure 2a is further shown in Figure 2c,d. Clearly, the constituent Ti powder and Ni powder distribute rather homogenously in the mixed powder.

### 3.2. Effect of Sintering Temperature on Pore Characteristics

**Figure 3:**
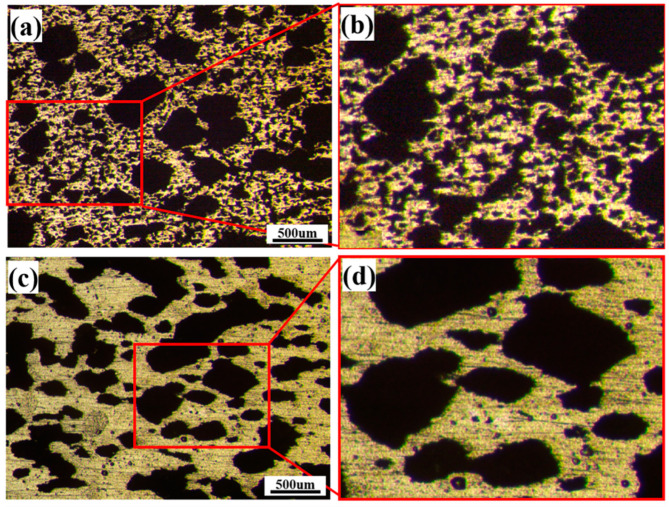
OM images of fabricated porous NiTi alloys sintered at different temperatures: (**a**,**b**) 950 °C; (**c**,**d**) 1000 °C.

**Figure 4:**
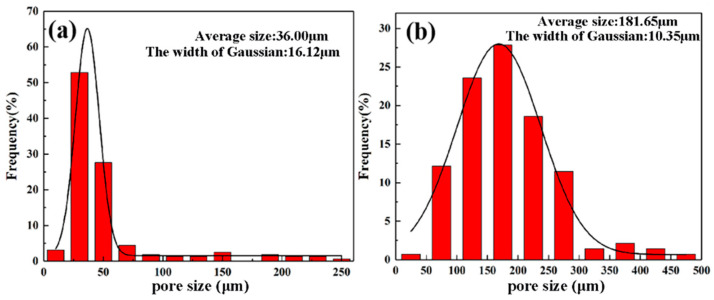
Pore size distribution maps of fabricated porous NiTi alloys sintered at different temperatures: (**a**) 950 °C; (**b**) 1000 °C.

Figure 3 presents OM images illustrating the pore morphology within the fabricated porous NiTi alloys at different sintering temperatures. The corresponding experimental results for density, porosity and pore size are shown in Table 1 and Figure 4. Obviously, the porous NiTi alloy sintered at 950 °C possesses an uneven pore distribution; namely, except for these large-sized pores, extensive minute pores can be found in the matrix (Figure 3a,b). Furthermore, it is worth noting that the edges of minute pores are relatively sharp. This phenomenon has also been reported in work by Zhu et al. [12], where they attribute this phenomenon to the insufficient diffusion of atoms resulting from an insufficient sintering temperature. Table 1 shows that the determined density of the fabricated porous NiTi alloy is 2.556 g/cm^3^ with a standard error of 0.27 g/cm^3^, and the determined porosity is calculated to be 60.4% with a standard error of 4.19%. Meanwhile, Figure 4a demonstrates that the determined average pore size is 36.00 μm with a Gaussian width of 16.12 μm. Actually, the width of the Gaussian distribution in Figure 4 is a statistical term which indicates the degree of dispersion in a data set, i.e., it shows the variation or dispersion in the mean or average value. A small value for the width of a Gaussian distribution indicates that the data points are closer to the mean value, while a large value for the width of a Gaussian distribution indicates that the data points are more dispersed. In comparison, small-sized pores vanish and large-sized pores distribute homogenously within the porous NiTi alloy sintered at 1000 °C (Figure 3c,d). The measured density, porosity and average pore size are obviously different from those of the porous NiTi alloy sintered at 950 °C, namely 3.030 g/cm^3^ with a standard error of 0.30 g/cm^3^, 51.8% with a standard error of 3.65% (Table 1) and 181.65 μm with a Gaussian width of 10.35 μm, respectively (Figure 4b).

Obviously, the sharp reduction in the average pore size (36.00 μm) in the porous NiTi alloy sintered at 950 °C is closely related to the formation of minute pores. The underlying mechanism of this formation is as follows: the element Ni has a relatively lower melting point and a lower evaporation energy compared to the element Ti. Therefore, the diffusion coefficient of a Ni atom would be higher than that of a Ti atom during the sintering process. This results in the formation of small cavities or pores (Kirkendall pores) in locations occupied primarily by Ni atoms [16,17]. Afterwards, these newly formed small pores tend to integrate into these pre-existing large-sized pores owing to the cavity concentration difference between small pores and large-sized pores [18]. It is clear that the integration stage of small pores into large-sized pores is not completed during sintering at 950 °C, as compared to the OM image of the porous NiTi alloy sintered at 1000 °C. This shows that the increase in the sintering temperature in the present study accelerates the integration stage of small pores into large-sized pores during the sintering process of fabricating porous NiTi alloys. 

As for the effect of the sintering temperature on the density and porosity of the fabricated porous NiTi alloys, many investigations [19,20,21] have reported that after sufficient diffusion of atoms, shrinkage plays an important role in the homogeneous distribution of the formed pores, as well as the reduction in pore size. Zhu et al. [18] attributed this phenomenon to a reduction in the Gibbs free energy, which leads to a decrease in the gas–solid interfacial area. Therefore, the increasing density and porosity in the porous NiTi alloy sintered at 1000 °C can be understood. 

### 3.3. Effect of Sintering Temperature on Microstructure Characteristics

**Figure 5:**
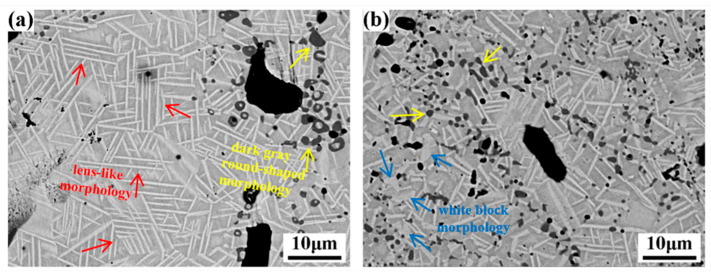
SEM maps of samples at different sintering temperatures: (**a**) 950 °C; (**b**) 1000 °C.

Figure 5 presents the SEM characterization results for the fabricated porous NiTi alloys at different sintering temperatures, where arrows with different colors show the existence of different precipitates. These SEM images, which were taken using backscattered electrons, clearly reveal the coexistence of secondary phases with distinctive morphologies, such as white lens, dark gray round-shaped and white block structures, within the sintered porous NiTi alloys. This demonstrates that there are no significant variations in the phase types within the porous NiTi alloys at different sintering temperatures. Specifically, in the sample sintered at 950 °C, a substantial number of lens-like phases are uniformly distributed throughout the matrix (Figure 5a). In contrast, in the sample sintered at 1000 °C, although these lens-like phases also distribute homogeneously within the matrix, their density is notably reduced (Figure 5b). These observations suggest that there are indeed variations in the phase distribution of porous NiTi alloys at different sintering temperatures.

**Figure 6:**
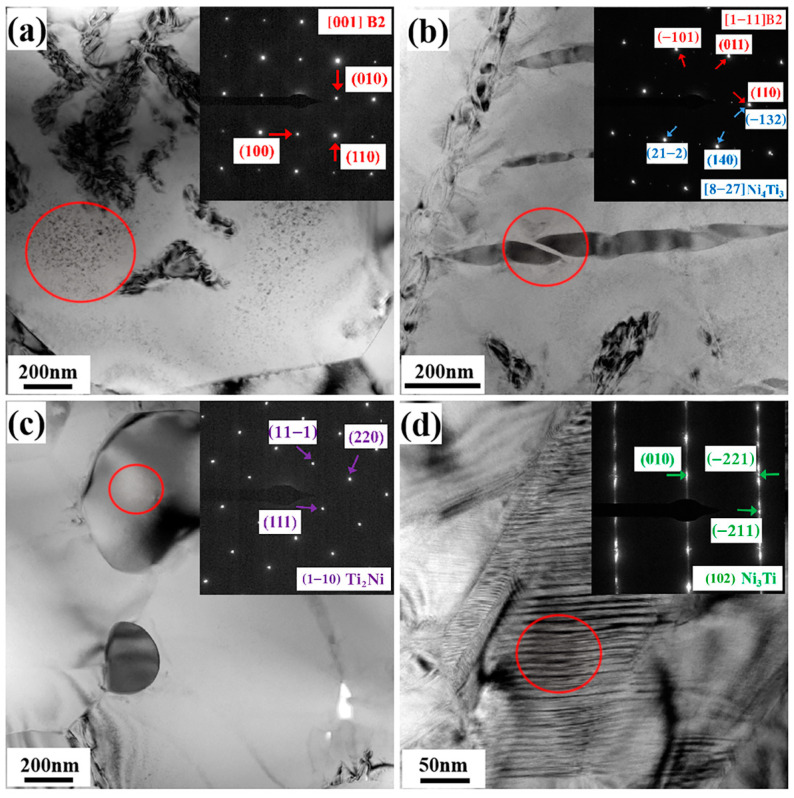
BF images and the corresponding SAEDPs of sintered porous NiTi alloy at 1000 °C: (**a**) matrix region with B2 NiTi; (**b**) region with the Ni_4_Ti_3_ phase; (**c**) region with the Ti_2_Ni phase; and (**d**) region with the Ni_3_Ti phase.

The identification of secondary phases in the porous NiTi alloy sintered at 1000 °C is further carried out using bright field (BF) images and the corresponding selected area electron diffraction patterns (SAEDPs), as depicted in Figure 6. These TEM results show that the matrix of the sintered porous NiTi alloy is B2 NiTi. Furthermore, the presence of the Ni_4_Ti_3_ phase, characterized by a lens-like morphology, is observed within the matrix, as depicted in Figure 6b. Figure 6c reveals the presence of the Ti_2_Ni phase, which displays a round-shaped structure with distinct interfaces. Notably, there is also an aggregated Ni-rich phase, namely the Ni_3_Ti phase, located in a localized region of the matrix. This phase exhibits typical stacking fault characteristics [22], as illustrated in Figure 6d. Obviously, the TEM observation results are sufficiently consistent with these SEM results, confirming the presence of various secondary phases within the fabricated porous NiTi alloys.

Zhu et al. [12] have reported potential reactions during the sintering process of fabricating porous NiTi alloy as follows: Ni + Ti → NiTi + 67 kJ/mol, Ni + Ti → Ti_2_Ni + 83 kJ/mol and Ni + Ti → Ni_3_Ti + 140 kJ/mol. Obviously, the Ti_2_Ni phase and the Ni_3_Ti phase are more thermodynamically favorable than the NiTi matrix. Ismail et al. [11] have reported that the Ti_2_Ni phase and Ni_3_Ti phase are rather stable, and they cannot be easily removed by changing the sintering temperature. This is the reason for the formation of the Ti_2_Ni phase and Ni_3_Ti phase in the fabricated porous NiTi alloys (Figure 5).

Jafar et al. [23] have reported that, as a typical Ni-rich intermetallic phase, Ni_4_Ti_3_ precipitates usually form during the thermomechanical treatment of Ni-rich NiTi alloys (Ni usually exceeds 50.5 at.%). The literature search in [10] summarizes that Ni_4_Ti_3_ precipitates cannot be observed within fabricated porous NiTi alloys during the sintering process of MWS, PS and spark plasma (SPS). Ismail et al. [11] reported the formation of Ni_4_Ti_3_ precipitates in a fabricated porous Ni-rich NiTi alloy (the Ni element is as high as 50.9 at.%) during the sintering process of MIM. However, in the present study, the Ni element is only 50.0 at.%, and the Ni_4_Ti_3_ phases are still formed during the sintering process of PS. Therefore, the formation mechanism of Ni_4_Ti_3_ precipitates in the present study should be investigated and declared clearly. Li et al. [24] documented that the enrichment of Ni content in the matrix and the absence of TLP lead to the formation of Ni_4_Ti_3_ precipitates during the sintering process. Actually, the diffusion rate difference between Ni atoms and Ti atoms would result in a high concentration of Ni at the early stage of the sintering process [18], and this contributes to the formation of Ni_4_Ti_3_ precipitates. Moreover, it is worth noting that elemental Ni powder and Ti powder are applied in the present study, and the maximum sintering temperature is 1000 °C. Li et al. [24] reported that a eutectic reaction (NiTi + Ni_3_Ti → TLP) would happen at 1118 °C. Although TLP only persists for a short period of time and would revert to a solid after further alloying, it has been proven to be helpful to the homogenization of the sintering composition. The obviously lower sintering temperature applied in the present study would result in the absence of TLP during the sintering process, and therefore solid-state diffusion with an obviously lower diffusion rate as compared to that of TLP would serve as the major contributor to the elimination of the Ni concentration. This would play a role in the further formation and multiplication of Ni_4_Ti_3_ precipitates. Moreover, as atom diffusion happens more quickly at a sintering temperature of 1000 °C, the concentration of Ni would be alleviated, as would the number of formed Ni_4_Ti_3_ precipitates within the fabricated porous NiTi alloys, as shown in Figure 5b.

## 4. Conclusions

In the present study, elemental powder sintering was applied to fabricate porous NiTi alloys with equal atomic ratios. To illuminate the effect of the sintering temperature on their pore characteristics and microstructure characteristics, sintering temperatures of 950 °C and 1000 °C were applied in the present study. By using characterization techniques, including OM, SEM and TEM, the following conclusions can be drawn.

(1) The porous NiTi alloys prepared at 950 °C and 1000 °C have densities of 2.556 g/cm^3^ and 3.030 g/cm^3^, porosities of 60.4% and 51.8% and average pore sizes of 36.00 μm and 181.65 μm, respectively. An increase in sintering temperature is useful to improve the densification of sintered porous NiTi alloys.
(2) There is no difference in the phase types within the fabricated porous NiTi alloys prepared at 950 °C and 1000 °C; namely, there are a large number of Ni_4_Ti_3_ precipitates with a lens-like morphology, a small amount of Ni_3_Ti precipitates with a round-shaped morphology and a small number of Ti_2_Ni precipitates with a block morphology in the B2 NiTi matrix.
(3) The phase distribution within the porous NiTi alloy sintered at 950 °C is obviously different from that within the porous NiTi alloy sintered at 1000 °C; that is, with an increase in the sintering temperature, the number of Ni_4_Ti_3_ precipitates decreases obviously. The formation of the Ni_4_Ti_3_ phase in the present study is closely related to the enrichment of Ni content in the matrix owing to the diffusion rate difference between Ni atoms and Ti atoms and the absence of TLP during the sintering process owing to the relatively low sintering temperature (lower than the eutectic temperature of 1118 °C). Compared to a sintering temperature of 950 °C, a sintering temperature of 1000 °C would speed up atomic diffusion, which contributes to a reduction in the enrichment of Ni as well as the number of formed Ni_4_Ti_3_ precipitates.

## Figures and Tables

**Table 1:** Specific data about density and porosity at different measurement positions of fabricated porous NiTi alloy.

Temperature	Measured Data	Location 1	Location 2	Location 3	Location 4	Location 5	Location 6	Location 7	Location 8	Average Value	Standard Error
950 °C	Density (g/cm^3^)	2.453	2.993	2.510	2.352	2.224	2.949	2.497	2.477	2.556	0.27
	Porosity (%)	62.0	53.6	61.2	63.5	65.5	54.4	61.2	61.7	60.4	4.19
1000 °C	Density (g/cm^3^)	2.550	3.080	2.896	3.061	3.190	3.082	3.201	3.202	3.030	0.30
	Porosity (%)	59.8	50.8	54.2	51.4	49.3	51.0	49.1	48.9	51.8	3.65

## Data Availability

Data are contained within the article.
